# Retrospective analysis of temporalis muscle thickness as a biomarker of functional level in adults with cerebral palsy

**DOI:** 10.1177/18758894261476165

**Published:** 2026-08-04

**Authors:** Hyun Iee Shin, Rajashree Srinivasan, Saylee Dhamdhere, Annie Abraham, Heakyung Kim

**Affiliations:** 1 Department of Rehabilitation Medicine, 26729 Chung-Ang University Hospital and College of Medicine, Seoul, Republic of Korea; 2 Department of Physical Medicine and Rehabilitation, 12334 University of Texas Southwestern Medical Center, Dallas, TX, USA

**Keywords:** adult cerebral palsy, GMFCS, temporalis muscle thickness, sarcopenia, cerebral palsy

## Abstract

This retrospective cohort study investigated the association between temporalis muscle thickness (TMT) and functional level in adults with cerebral palsy. TMT is a non-invasive radiographic measure that can be measured from brain magnetic resonance imaging (MRI) scans and used as a surrogate marker for sarcopenia to reflect whole body skeletal muscle mass. Adults with cerebral palsy (CP) are at increased risk of functional decline and sarcopenia. TMT has emerged as a potential biomarker of global muscle mass and nutritional status; however, its clinical relevance to functional mobility in adults with CP remains unclear. This study sought to investigate the association between TMT and ambulatory function in adults with CP. This retrospective cross-sectional study included adults with CP who underwent brain MRI. TMT was measured bilaterally and averaged. Ambulatory function and past medical histories were extracted from medical records. A total of 140 adults with CP were included. In multivariable analysis, greater TMT was independently associated with higher odds of ambulatory function (adjusted OR 1.31, p=0.037). Tube feeding was significantly associated with lower odds of being ambulatory (adjusted OR 0.13, p=0.016). TMT measured on routine brain MRI was independently associated with ambulatory function in adults with CP. TMT may provide complementary information to established clinical classifications.

## Introduction

Research focusing on adults with cerebral palsy (CP) remains limited compared with pediatric populations. As individuals with CP age, they encounter increasing healthcare challenges, including higher rates of chronic conditions, discontinuities in care during transition to adulthood, and limited guidance tailored to their needs.^1–4^ Objective and accessible approaches to functional assessment are particularly important in this population.

The Gross Motor Function Classification System (GMFCS) is a well-established and widely used tool to characterize motor function in individuals with CP, providing important insights into mobility and overall functional status. Although GMFCS levels are generally stable, motor function in adults with CP may vary due to aging-related and health-related factors.^5,6^ Therefore, there remains a need for reliable, objective biomarkers that complement clinical classification systems and enhance functional assessment in the adult CP population.

Temporalis muscle thickness (TMT), derived from routine brain magnetic resonance imaging (MRI) or ultrasonography, has emerged as a non-invasive surrogate marker of muscle mass in diverse clinical populations. In neurosurgical, stroke, and geriatric cohorts, reduced TMT has been associated with frailty, adverse functional outcomes, and mortality.^7–12^ These findings suggest that TMT may reflect clinically meaningful variations in muscle status. However, the relationship between TMT and functional status in adults with CP has not been previously examined.

This study aimed to examine the association between TMT and functional level in adults with CP and to determine whether TMT is independently associated with current ambulatory status. Understanding this relationship may help inform more personalized screening and intervention strategies for adults with CP.

## Methods

### Study design and cohort

This retrospective cohort study included adults diagnosed with CP who underwent brain MRI between January 2010 and May 2025 at a tertiary hospital. This retrospective study was approved by the Institutional Review Board of the University of Texas Southwestern Medical Center (IRB No. STU20250435).

### Participants

**Figure 1.**
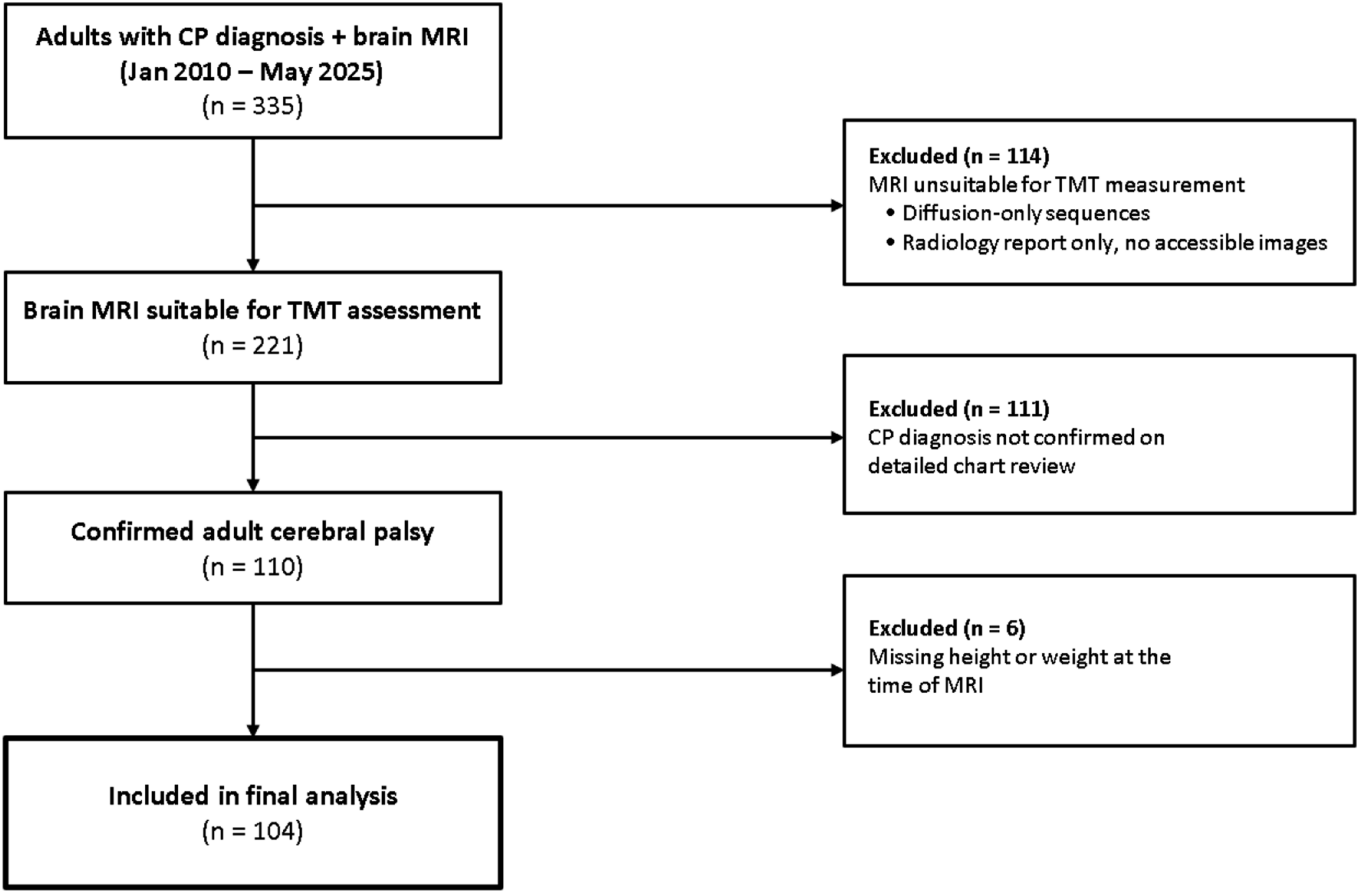
Flow diagram of participant selection. CP, cerebral palsy; GMFCS, Gross Motor Function Classification System; MRI, magnetic resonance imaging; TMT, temporalis muscle thickness.

Eligible participants were adults aged 18 years or older with documented CP diagnostic codes in the electronic medical record, in whom the diagnosis of CP was subsequently confirmed through detailed chart review by a board-certified physiatrist with expertise in adult CP care. In addition, participants were required to have a brain MRI containing T1-weighted axial images suitable for TMT measurement. Individuals were excluded if anthropometric data (height or weight) at the time of MRI were unavailable, or if a GMFCS level corresponding to the MRI period could not be retrieved from the medical record. Of 335 individuals initially identified, 104 met all eligibility criteria and were included in the final analysis (Figure 1).

### Clinical variables

Clinical variables extracted from medical records included age, sex, height, weight, body mass index (BMI), comorbidities, ambulatory status, and the date of brain MRI acquisition. GMFCS levels and CP subtypes were obtained from records documented within three months before or after the MRI examination. Ambulatory status was categorized based on GMFCS level: levels I–III were classified as ambulatory, and levels IV–V as non-ambulatory.^13,14^

### Measurement of TMT

**Figure 2.**
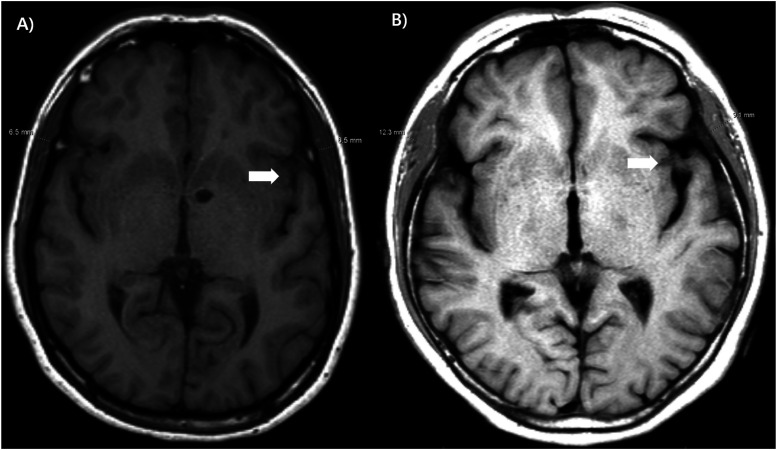
Temporalis muscle thickness (TMT) measurement. TMT was measured perpendicular to the long axis of the temporalis muscle on axial T1-weighted images using the Sylvian fissure (white arrow) as anatomical landmarks. Bilateral TMTs were measured. (a) Axial T1-weighted image from a non-ambulatory adult with cerebral palsy, (b) Axial T1-weighted image from an ambulatory adult with cerebral palsy.

TMT was measured manually by a single rehabilitation specialist (H.S.) with over 10 years of clinical experience. Measurements were performed on axial T1-weighted MR images, perpendicular to the long axis of the temporalis muscle. The orbital roof (cranio-caudal axis) and Sylvian fissure (anterior–posterior axis) were used as anatomical landmarks (Figure 2).^10,11^

Bilateral TMT values were averaged for analysis. Because bilateral motor involvement was common and a clearly unaffected side could not be reliably identified, the mean of both sides was used for the primary analysis.

### Statistical analysis

Continuous variables were compared between ambulatory and non-ambulatory groups using independent t-tests or Mann–Whitney U-tests, as appropriate. Categorical variables were compared using the chi-square test; Fisher’s exact test was applied when any expected cell count was less than five. The association between GMFCS level and TMT was evaluated using Spearman’s rank correlation coefficient.

Multivariable logistic regression was performed using the enter method, with age, sex, BMI, parenteral nutrition status, MRI year, and TMT included simultaneously as covariates based on clinical relevance. MRI year was included to adjust for potential confounding by calendar time across the 15-year study period. Adjusted odds ratios (ORs) with 95% confidence intervals (CIs) were reported.

All statistical analyses were conducted using JASP version 0.95.1 (University of Amsterdam, The Netherlands) and SPSS version 20 (IBM Corp., Armonk, NY, USA). Statistical significance was set at p < 0.05.

## Results

The mean age of the participants was 36.61 ± 13.46 years. Seventy five participants were ambulatory and 29 were non-ambulatory (Table 1). Ambulatory participants had greater mean weight, BMI, and TMT (p<0.05). Tracheostomy and parenteral nutrition rates were higher in non-ambulatory participants (p<0.05).

**Table 1.** Participant characteristics.

Variable	Total (n=104)	Ambulatory (n=75)	Non-ambulatory (n=29)	p-value
Age (year)	36.61 ± 13.46	36.51 ± 13.32	36.88 ± 14.03	0.948
Sex (Male)	39 (37.5%)	25 (33.3%)	14 (48.3%)	0.158
Height (m)	1.63 ± 0.11	1.64 ± 0.10	1.60 ± 0.12	0.090
Weight (kg)	71.69 ± 21.50	75.65 ± 20.87	61.44 ± 19.98	0.002^*^
BMI (kg/m^2^)	27.00 ± 7.46	28.24 ± 7.41	23.77 ± 6.67	0.006^*^
TMT (mm)	7.92 ± 2.60	8.42 ± 2.50	6.62 ± 2.44	0.001^*^
Hypertension	31 (29.8%)	20 (26.7%)	11 (37.9%)	0.260
Type 2 diabetes mellitus	15 (14.4%)	9 (12.0%)	6 (20.7%)	0.350
Dyslipidemia	20 (19.2%)	13 (17.3%)	7 (24.1%)	0.430
Mood disorder	51 (49.0%)	37 (49.3%)	14 (48.3%)	0.923
Cardiovascular disease	10 (9.6%)	5 (6.7%)	5 (17.2%)	0.137
Seizure	31 (29.8%)	22 (29.3%)	9 (31.0%)	0.897
Orthosis	4 (3.8%)	4 (5.3%)	0 (0.0%)	0.574
Intellectual disability	14 (13.5%)	8 (10.7%)	6 (20.7%)	0.206
Scoliosis	8 (7.7%)	5 (6.7%)	3 (10.3%)	0.683
Visual impairment	8 (7.7%)	6 (8.0%)	2 (6.9%)	1.000
Tracheostomy	4 (3.8%)	0 (0.0%)	4 (13.8%)	0.005^*^
Parenteral nutrition	10 (9.6%)	3 (4.0%)	7 (24.1%)	0.005^*^
GMFCS level				
Level 1	28 (26.9%)	28 (37.3%)	​	​
Level 2	23 (22.1%)	23 (30.7%)	​	​
Level 3	24 (23.1%)	24 (32.0%)	​	​
Level 4	20 (19.2%)	​	20 (69.0%)	​
Level 5	9 (8.7%)	​	9 (31.0%)	​
Type of CP				
Spastic	68 (65.4%)	48 (64.0%)	20 (69.0%)	​
Dystonic	6 (5.8%)	4 (5.3%)	2 (6.9%)	​
Athetoid	3 (2.9%)	1 (1.3%)	2 (6.9%)	​
Ataxic	2 (1.9%)	2 (2.7%)	0 (0.0%)	​
Mixed	25 (24.0%)	20 (26.7%)	5 (17.2%)	​

*p < 0.05.

Values are presented as mean ± SD or n (%).

BMI, Body Mass Index; TMT, Temporalis Muscle Thickness; GMFCS, Gross Motor Function Classification System; CP, Cerebral Palsy.

**Figure 3.**
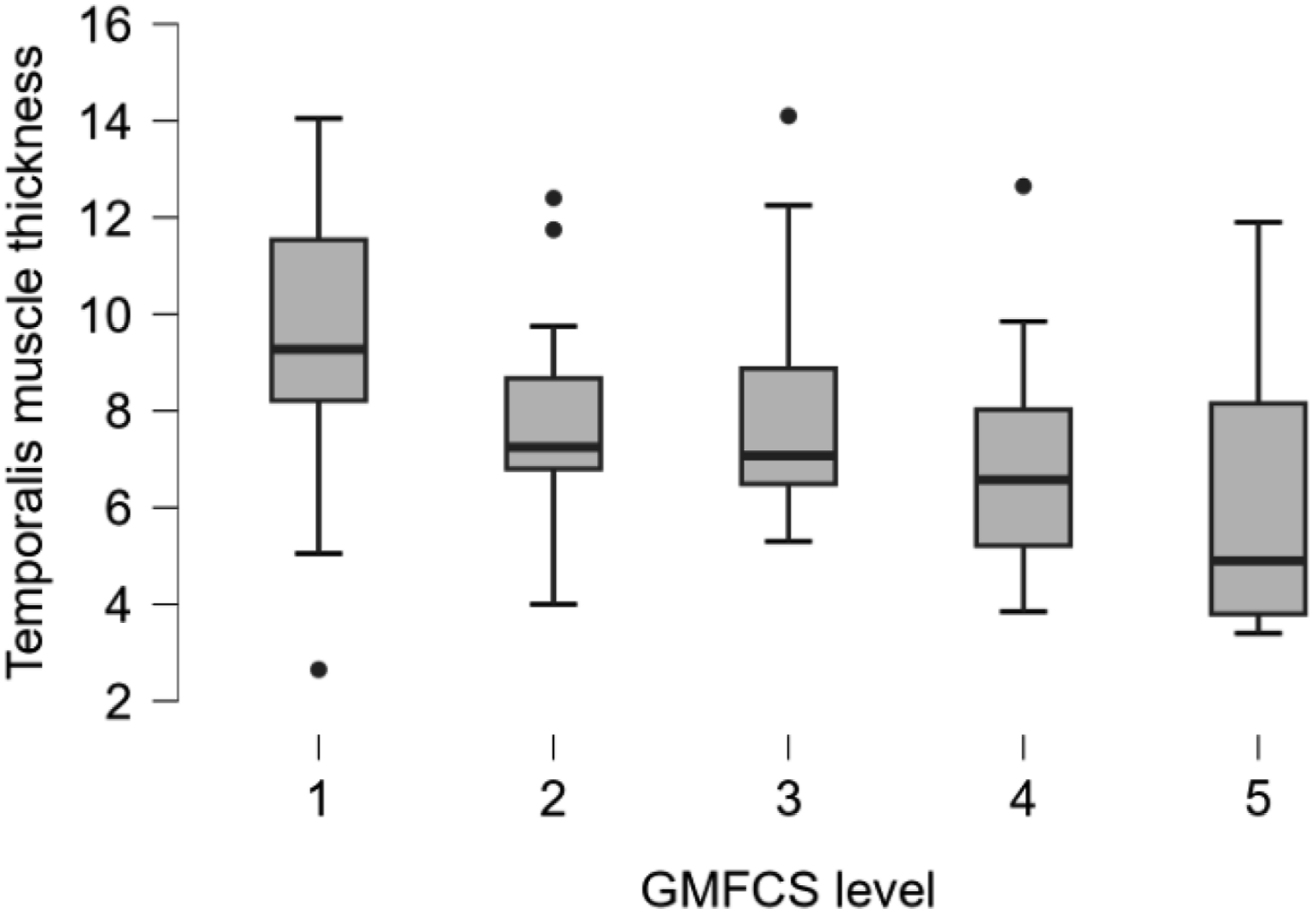
Temporalis muscle thickness according to GMFCS level. GMFCS, Gross Motor Function Classification System. Boxes represent the median and interquartile range. A significant negative association was observed between GMFCS level and temporalis muscle thickness (Spearman’s ρ = −0.41, p = 0.002).

TMT distributions across GMFCS levels are visualized in Figure 3 using box plots (median and interquartile range). There was a statistically significant negative correlation between GMFCS level and TMT (Spearman’s ρ = −0.402, p < 0.001), where higher GMFCS levels (indicating poorer motor function) were associated with reduced TMT.

In multivariable logistic regression analysis using the enter method, greater TMT was independently associated with higher odds of being ambulatory (adjusted OR 1.31, p=0.037) (Table 2). Tube feeding was significantly associated with lower odds of ambulatory function (adjusted OR 0.13, p=0.016). Age, sex, and BMI were not significantly associated with ambulatory status. The model was statistically significant (Δχ^2^ p=0.002), with Nagelkerke R^2^=0.264 and Tjur R^2^=0.200. MRI year, included to account for calendar time across the study period, was not independently associated with ambulatory status (adjusted OR 0.99, 95% CI 0.89–1.09, p = 0.811).

**Table 2.** Multivariable logistic regression analysis for factors associated with ambulatory level.

Variables	Adjusted OR (95% CI)	p value
Temporalis muscle thickness (mm)	1.31 (1.02–1.69)	0.037^*^
Sex (male)	0.55 (0.20–1.53)	0.258
Age (years)	0.98 (0.95–1.02)	0.384
BMI (kg/m^2^)	1.03 (0.94–1.13)	0.501
Parenteral nutrition	0.13 (0.03–0.68)	0.016^*^
MRI year	0.99 (0.89–1.09)	0.811

BMI, body mass index.

Adjusted odds ratios are from a multivariable logistic regression model with all listed variables entered simultaneously using the enter method. MRI year was included to adjust for calendar time across the 15-year study period.

*p < 0.05.

In the subgroup of participants with definite unilateral hemiplegia (n = 15), the affected side demonstrated significantly lower TMT values compared with the unaffected side (Wilcoxon signed-rank test, Z = −3.52, p < 0.001).

## Discussion

In this retrospective cohort of adults with CP, TMT emerged as a novel and independent biomarker of current functional status, as measured by GMFCS level. Ambulatory participants demonstrated significantly greater TMT, and in multivariable logistic regression, controlling for sex, age, BMI, parenteral nutrition status, and MRI year, greater TMT was independently associated with ambulation.

The present study adds to the growing evidence that TMT serves as a practical, reproducible biomarker across neurologic and chronic disease populations. Prior research has demonstrated TMT as a biomarker of sarcopenia, frailty, and poor functional outcomes in stroke, neuro-oncology, and geriatric cohorts.^7–12^ To the authors’ knowledge, this is the first study to evaluate TMT in adults with CP. While functional status can be clinically assessed through established classification systems and direct observation of mobility, these findings suggest that TMT may provide an additional, objective imaging-based correlate of current functional status. Incorporating TMT into routine MRI analysis or bedside ultrasonography protocols may therefore offer complementary information to support clinical assessment, particularly in settings where detailed functional evaluation is limited.

Adults with CP face unique barriers to healthcare access, including lower rates of outpatient visits and preventive care utilization compared to the general population.^15,16^ Socioeconomic vulnerability further limits opportunities to participate in regular physical therapy or rehabilitation programs once they transition out of pediatric care.^17^ In this context, reduced functional capacity due to pain, loss of muscle mass and flexibility, and early degenerative changes in the joints represent particularly critical concerns, yet opportunities for comprehensive assessment remain scarce. Transition programs play a critical role, as adults with CP often lose access to healthcare, therapy services, and school-based social participation when moving from pediatric to adult care. This loss can accelerate musculoskeletal complications and subsequent medical problems related to reduced activity, leading to early reduced functional capacity. Continuity of care, with early detection, timely interventions, and structured exercise, may help optimize muscle health and functional capacity in adults with CP. The findings suggest that TMT may serve as a practical complementary marker that can be drawn from imaging studies associated with functional status.^18,19^

Importantly, studies using dual-energy x-ray absorptiometry or bioelectrical impendence analysis (BIA) to assess body composition in adults with CP are scarce.^20,21^ BIA values are known to vary significantly with body position such as lying, sitting, or standing,^22,23^ suggesting that obtaining reliable measurements in adults with CP, who often present with spastic or rigid postures, may be particularly challenging. Furthermore, arm posture significantly impacts percent body fat measured by BIA,^23^ and the frequent presence of contractures or abnormal arm positioning in this population may further compromise accuracy. In such circumstances, TMT offers an indirect yet clinically meaningful method to capture aspects of muscle mass and functional reserve.^24^ Incorporating TMT into standard imaging protocols may therefore provide valuable information to guide individualized care in situations where more comprehensive evaluations are not possible.^18,19^

In this study, the association between TMT and functional status remained significant after adjustment for parenteral feeding in the multivariable model. While reduced masticatory activity may contribute to localized disuse-related atrophy, this finding suggests that diminished TMT cannot be explained solely by the absence of oral feeding. Rather, TMT may reflect broader physiological processes such as overall muscle mass status or functional activity. This interpretation is consistent with prior studies demonstrating that longitudinal reductions in TMT parallel insufficient energy and protein intake in older adults.^25^ On the other hand, even if the multivariable model was statistically significant, the explanatory capacity was modest from this study. It is also important to understand that ambulation in adults with CP reflects a complex interplay of multiple clinical and physiological factors. Ambulation in adults with CP is inherently multifactorial and influenced by neurological severity, muscle strength, spasticity, contractures, orthopedic deformities, pain, and cardiometabolic health.^26,27^ Interestingly, in individuals with unilateral hemiplegia, significantly lower TMT was observed on the affected side, supporting the contribution of side-specific muscle involvement and localized disuse. It should therefore also be acknowledged that TMT in adults with CP can reflect a multifactorial process involving localized disuse-related atrophy and broader systemic muscle wasting. Given the high prevalence of dysphagia and impaired chewing in non-ambulatory CP,^28,29^ diminished masticatory activity may also reasonably contribute to temporalis thinning in some patients. Future prospective studies incorporating objective assessments of oromotor function, swallowing capacity, and comprehensive nutritional evaluation will be necessary to further delineate the relative contributions of these mechanisms and to clarify the clinical utility of TMT in functional stratification and rehabilitation planning.

The developmental context should also be taken into account when interpreting muscle-related findings in adults with CP. Cerebral palsy is a lifelong neurodevelopmental condition characterized by immature brain injury and persistent motor impairment extending into adulthood.^30^ Several studies have shown that individuals with CP exhibit reduced muscle volume and altered muscle architecture from childhood, suggesting impaired muscle growth and failure to achieve normal peak muscle mass rather than purely age-related muscle loss.^31,32^ For example, imaging and histological studies have demonstrated smaller muscle cross-sectional area and shortened muscle fascicles in CP, consistent with developmental hypoplasia and altered muscle maturation. Therefore, reduced TMT in adults with CP should not be interpreted exclusively as a manifestation of age-related sarcopenia, as developmental hypoplasia may also play a significant role in diminished muscle thickness observed in this population. Still, as the current study examined functional differences within an adult CP cohort, the association between TMT and ambulation status may suggest that differences in muscle thickness are related to functional variation, although developmental factors likely contribute to baseline muscle characteristics.

Limitations of this study include its modest sample size and reliance on MRI-based TMT measurements, which may not be feasible in all clinical contexts. Nevertheless, TMT measurement by ultrasound has been validated and could provide a more accessible alternative for widespread implementation.^33^ Future prospective studies with larger sample sizes should further explore causal pathways, including whether improvements in muscle mass translate into measurable changes in TMT and functional status. This study was a retrospective study based on electronic medical records, which limited the ability to fully standardize certain measurements across all patients. In particular, height measurement in non-ambulatory individuals with CP can be challenging due to contractures and spinal deformities. However, in the study’s clinical setting, anthropometric measurements are routinely obtained by trained nursing staff as part of standard clinical care and recorded in the electronic medical record.

## Conclusion

In conclusion, TMT was independently associated with ambulatory functional status in adults with CP. The findings suggest that TMT could serve as a practical imaging marker associated with current functional status, potentially aiding in the recognition of functional limitation in this underserved population.
